# Combined Effect of Sow Parity and Terminal Boar on Losses of Piglets and Pre-Weaning Growth Intensity of Piglets

**DOI:** 10.3390/ani11113287

**Published:** 2021-11-17

**Authors:** Pavel Nevrkla, Jan Lujka, Tomáš Kopec, Pavel Horký, Radek Filipčík, Zdeněk Hadaš, Vendula Střechová

**Affiliations:** 1 Department of Animal Breeding, Faculty of AgriSciences, Mendel University in Brno, Zemědělská 1, 613 00 Brno, Czech Republic; jan.lujka@mendelu.cz (J.L.); tomas.kopec@mendelu.cz (T.K.); radek.filipcik@mendelu.cz (R.F.); zdenek.hadas@mendelu.cz (Z.H.); vendula.strechova@mendelu.cz (V.S.); 2 Department of Animal Nutrition and Forage Production, Faculty of AgriSciences, Mendel University in Brno, Zemědělská 1, 613 00 Brno, Czech Republic; pavel.horky@mendelu.cz

**Keywords:** parity of sows, terminal boars, losses of piglets, live-weight, average daily gain

## Abstract

**Simple Summary:**

The production of piglets is influenced by a range of factors; among the most important are parity of sows and genotype, which is half determined by terminal boars. In this study, reproductive performance, losses of piglets, and growth of piglets were compared depending on the parity and effect of terminal boars. The number of reared piglets, which is considered the most important parameter in the breeding of sows, was not affected by the evaluated factors. However, evaluation of pre-weaning losses of piglets clearly showed the lowest values in the third parity sows. Additionally, piglets of purebred Duroc and Large White_sirelinie_ boars showed the lowest losses before weaning and the highest growth intensity at the same time. In addition, the importance of studying interactions between factors was emphasized. These interactions revealed differences in some reproductive parameters and growth intensity of piglets in sows of a different parity after insemination with different terminal boars. The results show that the effect of terminal boars is crucial in the production of piglets. Based on the observed evaluation, the terminal boars of Duroc and Large White_sirelinie_ breeds can be recommended as optimal for the production of piglets.

**Abstract:**

This study analysed the effect of sow parity (P), terminal boars (TB), and their combination on reproductive parameters of sows, losses of piglets, and their individual live-weight (LW) and average daily gain (ADG) from birth to weaning. A total of 120 sows of Large White × Landrace hybrid combination from the first to the fourth parity (30 sows per parity) were included in the observation. The sows were inseminated by terminal boars of Pietrain (Pn), Large White_sireline_ (LW_SL_), and Duroc (D) breeds and Duroc × Large White_sirelinie_ (D × LW_SL_), Large White_sirelinie_ × Pietrain (LW_SL_ × Pn), and Duroc × Pietrain (D × Pn) hybrid combinations (20 sows per terminal boar population). The results proved a significant effect of P on the total numbers of piglets (TN), the numbers of stillborn piglets (SB), and the pre-weaning losses of piglets (L) (*p* ≤ 0.01) with the lowest losses found in the third parity sows. A significant effect of TB was confirmed for TN, the numbers of live-born piglets (LB), SB, the numbers of mummified piglets (M) and non-viable piglets (N), and (L) (*p* ≤ 0.01). The sows inseminated by D and LW_SL_ boars showed the lowest total numbers of piglets but also the highest survivability of piglets until weaning. Significant effects of interaction between P and TB were observed for the TN, SB, N, and L (*p* ≤ 0.05). Evaluation of growth parameters in piglets (*n* = 1547) showed that P significantly influenced LW in the first three weeks of life (*p* ≤ 0.01) and ADG from birth to Day 7 and from Day 15 to Day 21 (*p* ≤ 0.01). Additionally, a significant effect (*p* ≤ 0.01) of TB was proven on individual weight and ADG in all the observed time intervals, with the highest growth found in the piglets of the D and LW_SL_ boars. Significant interactions (*p* ≤ 0.01) between P and TB were found for LW and ADG in all the time intervals.

## 1. Introduction

The production of viable piglets of a good quality represents an elementary factor in the production of pigs for slaughter. Marandu et al. [1] and Jankowiak et al. [2] state that breeders’ endeavour is selection not only for the numbers of piglets born alive and reared per litter but also for an appropriate growth intensity of piglets in a litter. The authors emphasize that the heredity of birth weight in pigs is very favourable (with a coefficient around 0.31), unlike the heredity of reproductive performance parameters.

An important factor ensuring a profitable production of piglets is the optimal structure of sows in terms of parity. A high number of authors report optimal parameters of reproductive performance of sows and growth of piglets between the third and the fifth parity, and they conclude that these parities should represent the largest part of a herd [3,4,5]. The selection of suitable genotypes should be considered another crucial factor. The criteria for the selection of a suitable breeding combination are the results of the tests of hybrid populations. Their basic principle is the selection of genetically optimal populations for specific conditions. For producers of hybrid pigs, it is necessary to perform these tests in the conditions of production farms, as they represent the most important form of pig production [6]. The phenotypic level of reproductive parameters in sows, losses of piglets, and parameters of weight and growth intensity in piglets are also influenced by other factors: the effect of sows (or maternal genetic effect), the effect of season, farm, or the effect of feeding strategies [5,7,8,9]. The effects on growth intensity of piglets, mainly the effect of birth weight or sex or their combination on later growth intensity, have also been described [10,11]. Nevertheless, studies analysing the effect of terminal boars on the numbers of piglets born and reared and mainly on pre-weaning growth intensity of piglets are rare, even though their effect can be significant, as they represent 50% of the genetic source of piglets [12]. This effect is more described in association with parameters of carcass value and meat quality [13,14]. Some studies were focused on the combined effect of multiple factors on the prosperity of piglets. For example, Knecht et al. [5] analysed the combined effect of breed × season × parity. Nevertheless, the effect of parity and the effect of terminal boars have not yet been described in their interaction.

Therefore, the aim of this study was to analyse the effects of parity, terminal boars, and their combination on reproductive parameters of sows, losses of piglets, and individual weight and growth intensity of piglets expressed as average daily gain from birth to weaning.

## 2. Materials and Methods

Experimental observation took place in operational conditions of a production herd on a selected farm in the Czech Republic in accordance with the EU directive [15]. The experiment was carried out from February to July 2020.

### 2.1. Animals

A total of 120 F1 Large White × Landrace sows of the first to the fourth parity were included in the observation. The selection of the sows according to their parity and their distribution to individual populations of terminal boars was random.

Parities were balanced in the experimental population. Their effect was evaluated on 30 sows per parity. Gilts were first inseminated at the age of 235 ± 5 days. Prior to insemination, the oestrus of gilts was synchronized with Regu-Mate^®^ Porcine in order to enable the inclusion of the gilts in the reproductive process according to the principles of an all-in all-out system. All the sows included in the experiment were inseminated in February 2020. The same insemination method (intracervical insemination) was used for all the sows, performed by one insemination technician as one insemination and one re-insemination after 12 h. Farrowing was not induced in individual sows and occurred naturally during June and July 2020.

Terminal boars included in the study came from one insemination station in the Czech Republic and were bred in equal conditions. The boars were 20 ± 3 months old. Terminal boars used in the conditions of the Czech Republic for the production of final hybrids for fattening were included in the observation. These boars belonged to three pure breeds and three hybrid populations, namely Pietrain (Pn), Large White_sirelinie_ (LW_SL_), Duroc (D), Duroc × Large White_sirelinie_ (D × LW_SL_), Large White_sirelinie_ × Pietrain (LW_SL_ × Pn), and Duroc × Pietrain (D × Pn). Each breed or hybrid combination was represented by five boars (fathers). Five sows for each parity were randomly assigned to individual populations of the terminal boars. In total, there were 20 sows for the evaluated populations of terminal boars. The preparation of insemination doses and manipulation with them was equal for all the boars. A mixed ejaculate of five boars (fathers) of the individual populations of terminal boars was used (heterospermy). A two-step dilution of the ejaculate was used. Insemination doses were then transported to the farm at constant temperature (15–19 °C). Table 1 presents the distribution of sows for evaluation of the effects of parity and terminal boars and their combined effect on reproductive parameters of sows and losses of piglets.

All the reared piglets (*n* = 1547) were included in the evaluation of growth intensity.

Table 2 shows the numbers of piglets for the evaluation of the effects of parity and terminal boars and their combined effect on the growth of piglets.

Males were castrated on Day 4 after birth; at the same time, a single dose of iron (Uniferon^®^) was administered intramuscularly to the cervical muscle of all the piglets. Other vaccination or treatment procedures were not applied during the rearing of the piglets.

### 2.2. Evaluated Parameters

After birth, the piglets were marked with an individual numeric code. Selected reproductive parameters of sows, namely total numbers of piglets (TN), numbers of live-born (LB) and stillborn piglets (SB), numbers of mummified foetuses (M), non-viable piglets (NV) and reared piglets (R) per litter, and the losses of piglets (L) from birth to weaning were evaluated for individual populations of terminal boars. Non-viable were those piglets that died within 6 hours after birth without an evident cause, whose weight did not exceed 750 g. Losses of piglets from birth to weaning were expressed as difference in values between the live-born and the reared piglets.

Evaluation of productive parameters of piglets, i.e., individual live-weight (LW) (kg) from birth to weaning and subsequent evaluation of their growth ability determined by the average daily gain (ADG) (g/day) were recorded in weakly intervals. A digital hanging scale with an accuracy of 0.01 g was used for individual weighing of piglets performed within 24 h after birth and then every 7 days until weaning, carried out at an average age of 28 days. The average daily gain was calculated from the beginning and end weights of piglets in the monitored time intervals.

### 2.3. Housing and Nutrition

The sows were kept in conditions of a production farm. At the time of insemination and the following 30 days, they were stabled in individual boxes (length 210 cm and width 65 cm) with a differentiated floor (all-concrete floor vs. grates); each sow had its own feeding unit and a drinker. The gilts were fed 2.9 kg of the mixture per piece per day; the sows were fed 4–6 kg of the mixture per piece per day according to their condition. The sows were then (31st–108th day of gravidity) transferred to group pens with the floor differentiated into the bed (all concrete) and dunging place (concrete grates). The group pens were equipped with automatic feed dispensers and automatic drinkers according to the number of sows. The capacity of each pen was up to 60 sows. The floor area was 2.40 m^2^ per animal. Pregnant gilts were then fed 3 kg of the mixture per piece per day. Sows from the 31st to the 85th day of gravidity were fed 2.8 kg of the mixture per piece per day, and from the 86th to the 108th day of gravidity, they were fed 3.5 kg of the mixture per piece per day. One week before the expected farrowing, the sows were moved to the farrowing house, where they were stabled in individual farrowing boxes with farrowing cages. Feed dispensers and drinkers were available, and the feeding dose was calculated according to the number of piglets born. The basic dose for sows was 3 kg of mixture per day with a 0.5 kg addition per piglet. The length of the farrowing cage was 240 cm with a width of 70 cm. The overall length of the farrowing box was 240 cm with a width of 190 cm. The area per one piglet was 0.20 m^2^. The boxes were equipped with heating pads and infrared lamps for piglets. A drinker and feed for piglets were available in each box. The feed mixture was moistened for all sow categories. Piglets were fed with the dry feeding mixture ad libitum from the 5th day of age. The stable was equipped with an automatic ventilation system.

Table 3 presents the composition of the feed mixture for sows and piglets.

### 2.4. Statistical Analysis

The data were analysed in SAS 9.1 software [16]. A general linear model (PROC GLM, PROC GENMOD) was used for evaluation of the effect of individual factors. For reproductive and productive parameters, the statistical significance (at *p* ≤ 0.05 and *p* ≤ 0.01 level) of individual factors entering the model was assessed using TYPE III Sum of Squares. All statistically significant effects were included in both models; subsequent post hoc analysis evaluated the effect of parity and terminal boars according to the aim of this study.

#### 2.4.1. Reproductive Parameters of Sows

In the case of the reproductive parameters, the hypothesis of normal frequency distribution was rejected, and the parameters were analysed using the GENMOD procedure with an assumption of Poisson distribution. Significance of differences (*p* ≤ 0.05) between individual LSM (least square means) within parity and terminal boars was tested using the Chi-square test (Table 4).

Effect on reproductive parameters of sows was tested using the model equation:

y_ij_ = µ + α_i_ + β_j_ + (αβ)_ij_ + ε_ij_

y_ij_—the relevant dependent variable, µ—general mean (intercept), α_i_—the ith parity effect (j = 1–4), β_j_—the jth boar effect (i = 1–6), (αβ)_ij_—interaction of the ith parity effect and the jth boar effect, ε_ij_—random residual error.

#### 2.4.2. Productive Parameters of Piglets

Growth parameters of piglets showed a normal frequency distribution, which was analysed using the Kolmogorov–Smirnov test. This group of parameters was evaluated by the GLM procedure. Significance of the differences (*p* ≤ 0.05) between individual LSM (least square means) for the factors of terminal boar and parity was tested using the Tukey–Kramer method (Table 5 and Table 6).

Effect on growth parameters of piglets was tested using the model equation:

y_ijkl_ = µ + b × w + α_i_ + β_j_ + γ_k_ + δ_l_ + (αβ)_ij_ + (αγ)_ik_ + (βγ)_jk_ + ε_ijkl_.

y_ijkl_—the relevant dependent variable, µ—general mean (intercept), b × w—regression coefficient b expressing the relation between y and birth weight of piglet w, α_i_—the ith parity effect (j = 1–4), β_j_—the jth boar effect (i = 1–6), γ_k_—the kth sex effect (k = 1: boar, k = 2: gilt), δ_l_—effect of the lth total number of piglets (l = 1: 6–12 piglets, l = 2: 13–16 piglets, l = 3: 17–19 piglets, l = 4: 20–23 piglets), (αβ)_ij_—interaction of the ith parity effect and the jth boar effect, (αγ)_ik_—interaction of the ith parity effect and the kth sex effect, (βγ)_jk_—interaction of the jth boar effect and the kth sex effect, ε_ijkl_—random residual error.

The interactions of the total number of piglets and other factors, like the three- or four-factor interactions, were not statistically significant for the evaluated growth parameters of piglets, therefore they were excluded from the model. Data in the tables are presented as least square means and root mean square error (RMSE).

## 3. Results

### 3.1. Reproductive Performance of Sows and Losses of Piglets

Table 4 presents values of reproductive parameters and losses of piglets from birth to weaning per litter according to the parity of sows, terminal boar used, and interaction of these factors. Parity had a significant effect on TN, SB, and L before weaning (*p* ≤ 0.01). Our study showed an insignificant effect of parity on numbers of LB and R. The highest numbers of piglets were born to the third parity sows. On the contrary, the lowest values of TN were recorded in the first parities. The first parity sows had the lowest values of SB against the highest values in the third parity sows. The L values were lowest in the third parity sows while the highest losses were observed in the fourth parity sows.

The effect of terminal boars was significant for the TN, LB, numbers of SB, M, and NV, and for the L values from birth to weaning (*p* ≤ 0.01). The present study showed an insignificant effect of terminal boars on the R. The highest TN was recorded in sows inseminated by the D × LW_SL_ boars; in contrast, the lowest values were found in sows inseminated by the D boars. Evaluation of the LB showed the same trend. The study also proved the lowest SB after insemination by the D × Pn boar; in contrast, the highest SB was found after insemination by the purebred Pn boars. Zero values of M were recorded for the sows inseminated by the Pn and D boars; in contrast, the highest values were found in sows inseminated by the D × Pn boars. The number of NV within 6 hours after birth was the lowest in the sows inseminated by the D and LW_SL_ boars and the highest in sows inseminated by the hybrid LW_SL_ × Pn boars. Low L values were recorded in piglets of the D, LW_SL_, and LW_SL_ × Pn boars, while the highest values of L were recorded in piglets of the D × LW_SL_ boars.

A significant effect of interaction between parity and terminal boar was recorded for the TN (*p* ≤ 0.05) with the highest values recorded in the third parity sows inseminated by the Pn boars and the lowest values found in the first parity sows inseminated by the LW_SL_ boars. Significant interaction effects were also found for the SB (*p* ≤ 0.01) with the highest value recorded in the third parity sows inseminated by the Pn boars against zero values found in the third parity sows inseminated by the LW_SL_ and D boars. Other significant interaction effects were observed for the NV (*p* ≤ 0.05), with the highest value found in the second parity sows inseminated by the LW_SL_ × Pn boars, while the lowest values were recorded in the second parity sows inseminated by the LW_SL_ boars. The L values were also influenced by the interaction between parity and terminal boar (*p* ≤ 0.01), with the highest L recorded in the fourth parity sows inseminated by the D × LW_SL_ boars and the lowest L found in the first parity sows inseminated by the LW_SL_ and the second parity sows inseminated by the LW_SL_ × Pn boars.

### 3.2. Growth Ability of Piglets

Table 5 shows the LW of piglets in relation to the parity of sows, terminal boars used, and the interaction of these factors. The parity of sows significantly influenced the LW of piglets in the first three weeks of life (*p* ≤ 0.01). Analysis of individual birth LW of piglets showed the highest values in the fourth parity sows and the lowest in the third parity sows. On Day 7, the highest LW was recorded in the piglets of the second parity sows and the lowest in the piglets of the third parity sows. The same trend was observed in piglets at the age of 14 days.

A significant effect (*p* ≤ 0.01) of terminal boars was proven on LW in all the observed time intervals. Analysis of individual birth weight (BW) showed the highest values in piglets of the D × LW_SL_ boars against the lowest values in piglets of the D boars. On Day 7, the highest LW was recorded in piglets of the D boars and the lowest weight in piglets of the LW_SL_ × Pn boars. A similar trend was repeated in the observed intervals until weaning.

Significant effects of interactions (*p* ≤ 0.01) between parity and terminal boars were recorded for the LW in all the monitored time intervals. The highest LW at birth was observed in pigs of the fourth parity sows inseminated by the Pn boars, while the lowest birth weight was found in the piglets of the third parity sows inseminated by the D boars and in the piglets of the first parity sows inseminated by the D × Pn boars. The highest LW on Day 7 was found in piglets of the first parity sows and the D boars; in contrast, the lowest LW was recorded in the piglets of the third parity sows inseminated by the LW_SL_ × Pn boars. At the age of 14 days, the highest LW was found in the piglets of the first parity sows and the D boars and the lowest in the piglets of the first parity sows and the D × LW_SL_ boars. The same trend was observed on Day 21 and Day 28.

Table 6 shows the ADG of piglets in relation to the parity of sows, terminal boars used, and interaction of these factors. Evaluation of the ADG showed that during the first week of life, piglets of the second parity sows achieved the highest gains, while the piglets of the third parity sows achieved the lowest gains. From Day 15 to Day 21, the trend was the opposite.

A significant effect (*p* ≤ 0.01) of terminal boars was proven on ADG in all the observed time intervals. The piglets of the D boar reached the highest ADG in the first week of life, while the lowest value was observed in the piglets of the LW_SL_ × Pn boars. In the second week, the highest ADG was recorded in the piglets of the D boars against the lowest value found in the piglets of the D × LW_SL_ boar. This trend was repeated in the third week of life. However, in the fourth week, the growth of piglets of the D boars slowed down and the highest ADG was recorded in the piglets of the hybrid LW_SL_ × Pn boars. Nevertheless, evaluation of the ADG from birth to weaning showed the highest values in piglets of the D boar against the lowest values in piglets of the D × LW_SL_ boars.

Significant effects of interactions (*p* ≤ 0.01) between parity and terminal boars were recorded for the ADG in all the monitored time intervals. The highest ADG in the first week of life was recorded in the piglets of the first parity sows and the D boars, while the lowest was observed in the piglets of the same parity sows and the D × Pn boars. In the second week of life, the highest ADG was also achieved by the piglets of the first parity sows and the D fathers, while the lowest was achieved by the piglets of the same parity sows and the D × LW_SL_ boars. During the third week, the ADG was highest in the piglets of the third parity sows and the D boars and the lowest in the piglets of the fourth parity sows and the Pn boars. The fourth week of life was characterized by the highest ADG in the piglets of the third parity sows and the LW_SL_ fathers against the lowest ADG in the piglets of the same parity sows and the D boars. The analysis of ADG (birth–weaning) revealed the highest values in piglets of the first parity sows inseminated by the D boars and the lowest values in the same parity sows inseminated by the D × LW_SL_ boars.

## 4. Discussion

### 4.1. Reproductive Performance of Sows and Losses of Piglets

In the present study, parity significantly affected the values of TN, SB, and L. The LB and R were not influenced by parity. These results can be related to the fact that most studies evaluating the effect of parity compared pure breeds, while hybrid sows were studied only rarely. Our findings correspond to the results of Segura et al. [17], who evaluated reproductive performance in F1 sows (Large White × Landrace) from the first to the sixth parity, which confirms that current hybrid sows are characterized by higher uniformity in reproductive parameters, regardless of parity. However, this trend was the opposite in pure breeds with significant differences in the number of live-born and reared piglets [5,18,19]. All the studies mentioned above are in accordance with our findings that the first parity sows had the lowest total numbers of piglets when compared to later parities. This finding is probably associated with the completion of growth and subsequent increase of the capacity of the uterus [20], as there were no significant differences observed between the second and the third parity. According to our findings, the highest SB was recorded in the third parity sows, which is probably related to the highest total number of piglets born [21]. Škorjanc et al. [22] and Nevrkla et al. [23] proved significant positive correlations between the litter size and the number of stillborn piglets. Evaluation of the losses of piglets from birth to weaning showed the highest values in the fourth parity sows. Hagan and Etim [24] also proved the highest pre-weaning mortality in piglets of the fourth parity sows. These results might be associated with the size of the sows. The fourth parity sows grow old and heavier and become less agile, and the risk of overlying piglets increases when compared to younger energetic sows.

The effect of terminal boars on the reproductive performance of sows and the losses of piglets has been studied to only a limited extent. However, the results of this study show that this factor has a significant effect on most of the observed parameters. According to Jankowiak et al. [2], the genotype is an important factor affecting the reproductive parameters in sows. The authors add that the genotype of piglets, thus the breed or hybrid combination used in paternal position, has a much more limited effect. In contrast to these statements are the conclusions of McCann et al. [12], who proved that the breed in terminal position affects some reproductive parameters in the litter. Specifically, F1 sows (Large White × Landrace) inseminated by the D and D × LW reached more live-born piglets than the sows inseminated by the LW and LW × Pn boars, by more than one piglet. Furthermore, the sows inseminated by the Pn boars had 0.7 stillborn piglets more than the sows inseminated by the D boar (*p* < 0.05). The mortality of piglets from birth to weaning was also higher by 4% after insemination by the Pn boar. Pedersen et al. [6] reported 0.5 live-born piglets more after insemination by the Pn boar than by the D boar; however, pre-weaning mortality was also higher in the piglets of the Pn boars, by more than 0.5 piglet, which corresponds to our results. As in the present study, Magowan and McCann [12] did not confirm the effect of terminal boars on the number of piglets reared; however, they found a significant effect (*p* < 0.01) on losses of piglets from birth to weaning. Interestingly, they recorded differences between two populations of the Pietrain breed, Austrian and Belgian (26.79% vs. 15.94%), which indicates two distinct breeding lines of the populations evaluated. The authors add that genetic variability of the original populations in various selection combinations can have a different effect on the phenotypic expression of sows and survivability of piglets, which is evident also from the results of our study. The documented differences in the total number of piglets born to the observed terminal boars can be related to the quality of ejaculate, which can vary significantly depending on the breed [25,26]. A smaller size of a litter at birth can be caused by higher foetal mortality. According to Hagan and Etim [24], lower numbers of zygotes can result from inferior semen quality in boars. Rodriguez et al. [27] suggest that boar lines with faster growth can have worse quality of ejaculate that can affect litter size, which could be an explanation for the lowest reproductive parameters found in the sows inseminated by the D boars, while the growth rate of their piglets was the highest. Nevertheless, further studies are needed. Terminal boars can affect the occurrence of stillborn piglets indirectly, depending on the number of eggs fertilized and subsequently on the number of viable foetuses. It is known that the higher the litter size, the higher probability of stillbirth [28,29]. A study conducted by Opressing et al. [30] suggests that there are differences between the breeds in sensitivity to Porcine circovirus type 2 (PCV 2), which can be associated with the occurrence of mummified or less viable piglets. Nathues et al. [31] and Ellegaard et al. [32] state that the Danish population of the Duroc breed is selected to reduce the occurrence of the PCV 2. These studies indirectly point to genetic dispositions that can influence the occurrence of mummified or non-viable piglets; however, more studies are needed.

The effects of interaction between the parity of sows and terminal boar used have not been described yet. Our observation proved significant effects of interaction, specifically on TN and subsequently on the number of SB, NV, and pre-weaning L. The results show that terminal boars applied in the production of piglets can significantly influence the number of piglets born, depending on ejaculate quality, or the numbers of fertilized eggs and cleaving embryos [24]. This process significantly involves parity of sows, with higher reproductive potential recorded in sows of the second and following parities, due to completed growth [33]. It is evident that the paternal effect in combination with the parity of sows can be crucial for the survivability of piglets; however, the effects are uneven. This finding can be related to the variable strength of the heterosis effect [34], which is more pronounced the more different the original breeds are.

### 4.2. Growth Ability of Piglets

The present study showed that the parity of sows has an effect on the live-weight (LW) of piglets at birth and then at the age of 7 days and 14 days. The live-weight at 21 days of age and at weaning was not affected by parity. ADG was influenced by the parity in the first and the third week of life. ADG was also influenced by parity in the first and the third week of life. No significant effect of parity was found on ADG from birth to weaning. The results of most studies correspond to our finding that parity has an effect on the LW of piglets at birth [24,35]. Other studies [5,36] state that parity also influenced the LW at weaning, which was not confirmed in our observation. Klimas et al. [19], who studied the growth of piglets depending on the parity in the sows of three breeds (Lithuanian White, Landrace, and Large White), found no significant effect of parity on the weaning weight of piglets in any of the evaluated breeds. Same as in our observation, even in the studies mentioned above, the results of individual parities are uneven, which suggests that the growth of piglets can depend on other factors, such as season, breeding systems, and feeding strategies for sows and piglets, but also on genetic factors [5,19,35,37]. In some studies, the results of pure breeds are presented, and the results of hybrids are presented in other studies, while the weight of piglets is more homogeneous in hybrid sows than in the purebred sows [5]. Škorjanc et al. [22] state that the higher the number of piglets in the litter, the lower their LW, which could explain the lowest live-weight of piglets of the third parity sows, found in our study. This was subsequently reflected in the LW and ADG in the first weeks of life. From the third week of life, the differences among parities are minimal. Zotti et al. [11] describe significant, but uneven, differences in the growth intensity of piglets among the individual parities. The authors recorded the highest LW of piglets in the second parity sows (1.44 kg); in the first, third, and fourth parity, it was 1.33 kg. These values corresponded to the LW on the seventh day of age, with 2.51 kg in the second parity sows and 2.31–2.36 in the other parities. Nevertheless, from the 21st day of life, the differences in LW and ADG were not significant, which suggests that the environmental factors start to predominate during the weaning period.

Studies evaluating the effect of terminal boars on the pre-weaning growth ability of piglets are rare. Our analysis proved a significant effect of terminal boars on growth ability. Attention should therefore be paid to their selection. Evidently, the genetic basis of animals or different strategies of selection of the original breeds, emphasized by Jiang et al. [38], affect growth ability unevenly, which is apparent mainly for the D × LW_SL_ boars with the highest total number of piglets and the highest birth weight of piglets with average pre-weaning growth intensity at the same time. On the contrary, the lowest total number of piglets together with the lowest birth weight was recorded for the D boars; however, the growth intensity of piglets apparently increased, and they reached the highest weaning weight. Similar to our experiment, Cechova et al. [10] proved the effect of terminal boars on the individual birth weight of piglets (*p* < 0.01). The authors studied the effect of four terminal boars, LW_SL_, D, and Pn × Hampshire and D × Hampshire hybrids. They found the highest birth weight in piglets of the LW_SL_ boar (1.34 kg) against the other piglets, whose weight did not exceed 1.25 kg. Magowan et al. [13] also observed the effect of terminal boars on the weight of piglets at birth and at weaning (*p* < 0.01). Piglets of the Austrian Pn (APn) showed a higher birth weight (1.47 kg) than piglets of the Belgian Pn (BPn), Landrace, and LW_SL_ (1.59 kg). The highest weaning weight was found in the piglets of the LW_SL_ boar (9.12 kg) and the lowest in the piglets of the BPn boar (8.72 kg). Craig et al. [39] also evaluated the weights of piglets of various hybrid combinations at weekly intervals. They observed significant differences (*p* < 0.01) between the breeding combinations of piglets and emphasize that these differences between populations may reach up to 1 kg in individual intervals. The study of Škorjanc et al. [22] revealed that a weight significantly below 3 kg at the age of 14 days can be considered problematic, while a weight of 5 or more kilograms is considered above standard. Their observation showed that the terminal boar can influence the growth intensity of piglets by 15%. In contrast, Jankowiak et al. [2] state that the terminal boar can influence the birth weight of piglets, while further growth is influenced mainly by environmental factors. However, in this context, they admit that the genetic basis passed onto the piglets by boars can determine sensitivity to stress as a reaction to various external factors (manipulation with piglets, noise, etc.), which may affect their growth intensity indirectly, as confirmed also by Panzardi et al. [40]. Magowan et al. [13] did not prove any effect of terminal boars on the rearing weight of piglets; however, they consider this effect significant in the later stages of fattening.

The present study showed that the two-factor interaction of parity and terminal boar can have an important effect on the growth ability of piglets, as all the observed parameters were significantly affected by their combination. Growth parameters were influenced by both the parity of sows and terminal boar or genotype of piglets, as well as their interaction. Nevertheless, the interaction effects are often limited and do not manifest equally in all the hybrid combinations of pigs [24,41]. Our results show an evident trend that the piglets of the first parity sows inseminated by the D boars were characterized by the highest LW at weaning and the highest ADG (birth–weaning). The observed differences may be related to the fact that sows of different parities are characterized by different feed intakes, affecting their milk production, which has an effect on the growth of piglets. Variability in milk production in combination with the genetic basis of piglets determined by the terminal boars show significant effects on weight gains [42]. Knecht et al. [5] also documented a significant effect of genotype × parity interaction on the LW of piglets at birth and at weaning.

## 5. Conclusions

The results of the observation show that the structure of sows in terms of parity and selection of the terminal boars have a significant effect on piglet production, as evidenced by the significant interaction effects between parity and terminal boars. Results of the reproductive performance of sows suggest that the hybrid sow populations bred currently in the Czech Republic show lower variability in reproductive performance among parities. The experiment also revealed the lowest losses of piglets in the third parity sows. The highest number of live-born piglets was found in the hybrid boars of D × LW_SL_; nevertheless, these boars were also characterized by the highest losses of piglets and the lowest growth intensity of piglets. The lowest pre-weaning losses of piglets were recorded in the purebred LW_SL_ and D boars together with the highest growth intensity. Besides the main factors, the study also proved significant interaction effects of parity and terminal boars used, both in reproductive parameters and in the growth of piglets. These interactions demonstrate that the sows of individual parities reply differently to different terminal boars, which was apparent mainly in the numbers of stillborn piglets, non-viable piglets, and losses of piglets from birth to weaning, with the most favourable values found in the first parity sows inseminated by D and LW_SL_ boars. The highest growth ability of piglets was recorded for the D and LW_SL_ in sows of all parities, which confirms that these boars can be recommended as optimal for production of piglets.

## Figures and Tables

**Table 1:** Distribution of animals for analysis of reproductive parameters of sows and losses of piglets.

	Terminal Boars	Pn	LW_SL_	D	D × LW_SL_	LW_SL_ × Pn	D × Pn
Parity	Sows (*n*)	20	20	20	20	20	20
1	30	5	5	5	5	5	5
2	30	5	5	5	5	5	5
3	30	5	5	5	5	5	5
4	30	5	5	5	5	5	5

**Table 2:** Distribution of animals for analysis of growth of piglets.

		Terminal Boars	Pn	LW_SL_	D	D × LW_SL_	LW_SL_ × Pn	D × Pn
		Sows (*n*)	20	20	20	20	20	20
Parity	Sows (*n*)	Piglets (*n*)	257	258	244	272	241	275
1	30	338	56	51	62	53	60	56
2	30	401	69	67	57	87	62	59
3	30	402	70	68	62	65	64	73
4	30	406	62	72	67	67	55	87

**Table 3:** Composition of feed mixture for sows and piglets.

Ingredients (%/kg)	Inseminated and Pregnant Sows	Sows in Farrowing House	Suckling Piglets
Wheat	27	26	12
Barley	42	37.5	30
Corn	-	12	15
Oat	12	-	-
Wheat bran	9	-	-
Soybean meal	2	18	22
Rapeseed extracted meal	5	-	-
Rapeseed oil	-	2.5	-
Oatmeal	-	-	6
Lactose	-	-	0.5
Fish meal	-	-	5.5
Potato protein	-	-	3
Seaweed meal	-	-	1
Milk thistle seed	-	-	1
Minerals and vitamins	3	4	4
ME (MJ/kg) ^1^	11.74	13.18	13.25

^1^ ME, metabolizable energy.

**Table 4:** The effect of parity and terminal boar on selected reproductive parameters of sows and losses of piglets (pcs/litter).

Factor	Item	Total Number of Piglets	Live-Born Piglets	Stillborn Piglets	Mummified Piglets	Non-Viable Piglets ^1^	Reared Piglets	Losses of Piglets
**Effect of Individual Factors**								
Parity (P)	1	13.64 ^a^	12.43	0.94 ^a^	0.06	0.25	11.87	0.56 ^a^
	2	15.96 ^b^	14.08	1.50 ^a^	0.10	0.38	13.43	0.64 ^a^
	3	16.88 ^b^	13.94	2.40 ^b^	0.33	0.54	13.54	0.40 ^a^
	4	15.96 ^b^	14.08	1.46 ^a^	0.14	0.40	12.72	1.36 ^b^
Terminal boars (TB)	Pn	16.60 ^a,c^	13.56 ^a^	2.79 ^b^	0.01 ^b^	0.25 ^a,b^	12.47	1.09 ^b,c,d^
	LW_SL_	14.68 ^b,c^	13.00 ^a^	1.55 ^a^	0.30 ^ab^	0.14 ^a^	12.81	0.19 ^a^
	D	13.75 ^b^	12.29 ^a^	1.35 ^a^	0.01 ^b^	0.12 ^a^	11.98	0.31 ^a^
	D × LW_SL_	17.79 ^a^	16.11 ^b^	1.26 ^a^	0.08 ^ab^	0.41 ^a,b^	14.34	1.77 ^b,d^
	LW_SL_ × Pn	15.47 ^a,b^	13.18 ^a^	1.41 ^a^	0.12 ^ab^	0.83 ^b^	12.59	0.59 ^a,c^
	D × Pn	15.35 ^a,b^	13.66 ^a^	1.09 ^a^	0.48 ^a^	0.62 ^a,b^	13.15	0.51 ^a,c^
**Effect of Interaction Between the Factors**								
1	Pn	14.08 ^b^	11.67	2.00 ^b^	0.01	0.41 ^b^	11.26	0.41 ^b^
	LW_SL_	10.77 ^c^	10.70	0.01 ^c^	0.02	0.06 ^c^	10.63	0.07 ^c^
	D	13.05 ^c^	12.97	0.01 ^c^	0.02	0.07 ^c^	12.89	0.08 ^c^
	D × LW_SL_	16.88 ^a^	14.39	2.23 ^b^	0.06	0.24 ^b,c^	12.31	2.08 ^a^
	LW_SL_ × Pn	14.83 ^b^	13.94	0.63 ^c^	0.06	0.24 ^b,c^	13.67	0.27 ^b^
	D × Pn	12.19 ^b^	10.92	0.79 ^c^	0.17	0.49 ^b^	10.44	0.48 ^b^
2	Pn	15.48 ^b^	14.40	1.19 ^b,c^	0.03	0.10 ^c^	13.11	1.29 ^a^
	LW_SL_	14.95 ^b^	13.59	1.40 ^b^	0.39	0.03 ^c^	13.21	0.37 ^b^
	D	12.55 ^c^	11.90	0.60 ^c^	0.01	0.04 ^c^	11.66	0.24 ^b^
	D × LW_SL_	20.52 ^a^	17.90	2.00 ^b^	0.01	0.61 ^b^	16.49	0.41 ^b^
	LW_SL_ × Pn	16.41 ^a^	11.89	2.99 ^b^	0.18	1.54 ^a^	11.96	0.07 ^c^
	D × Pn	17.22 ^a^	14.79	0.82 ^c^	0.05	0.19 ^c^	13.17	1.62 ^a^
3	Pn	21.47 ^a^	14.13	5.61 ^a^	0.03	0.79 ^b^	13.21	0.93 ^b^
	LW_SL_	17.27 ^a^	14.69	2.40 ^b^	0.40	0.19 ^c^	14.30	0.38 ^b^
	D	14.99 ^b^	12.26	2.79 ^b^	0.02	0.07 ^c^	12.14	0.13 ^c^
	D × LW_SL_	16.11 ^a,b^	15.34	0.62 ^c^	0.14	0.14 ^c^	14.38	0.96 ^b^
	LW_SL_ × Pn	14.23 ^b^	12.10	1.21 ^b,c^	0.03	0.91 ^a,b^	11.17	0.32 ^b^
	D × Pn	17.22 ^a^	15.15	0.77 ^c^	1.33	1.33 ^a^	15.46	0.31 ^b^
4	Pn	15.36 ^b^	14.05	1.37 ^b,c^	0.06	0.04 ^c^	12.32	1.78 ^a^
	LW_SL_	15.73 ^b^	13.01	2.39 ^b^	0.38	0.33 ^b,c^	13.09	0.08 ^c^
	D	14.43 ^b^	12.03	1.98 ^b^	0.04	0.43 ^b^	11.22	0.81 ^b^
	D × LW_SL_	17.65 ^a^	16.82	0.20 ^c^	0.01	0.63 ^b^	13.19	3.63 ^a^
	LW_SL_ × Pn	16.42 ^a^	14.79	0.80 ^c^	0.21	0.62 ^b^	12.97	1.83 ^a^
	D × Pn	16.18 ^a,b^	13.76	1.98 ^b^	0.36	0.45 ^b^	13.53	0.23 ^b^
RMSE		2.95	2.67	1.37	0.45	0.64	2.66	0.83
*p*-value	P	**	NS	**	NS	NS	NS	**
	TB	**	**	**	**	**	NS	**
	P × TB	*	NS	**	NS	*	NS	**

^1^: Piglets that died within 6 h after birth for no evident reason. Pn: Pietrain. LW_SL_: Large White_sireline_. D: Duroc. D × LW_SL_: Duroc × Large White_sireline_. LW_SL_ × Pn: Large White_sireline_ × Pietrain. D × Pn: Duroc × Pietrain. Results of the variance analysis are indicated as significant (* *p* ≤ 0.05, ** *p* ≤ 0.01) or not significant (NS). ^a,b,c,d^ Mean values in the same column marked with a different superscript indicate statistical significance (*p* ≤ 0.05). Mean values with no superscript are not significantly different from any other values. RMSE: root mean square error.

**Table 5:** The effect of terminal boar and parity of sows on individual weight of piglets.

Factor	Item	Live-Weight (kg)				
		At Birth	Day 7	Day 14	Day 21	Day 28
**Effect of Individual Factors**						
Parity (P)	1	1.40 ^a^	2.74 ^a^	4.50 ^a,b^	6.27	7.56
	2	1.40 ^a^	2.80 ^a^	4.57 ^a^	6.33	7.65
	3	1.33 ^b^	2.61 ^b^	4.41 ^b^	6.23	7.55
	4	1.42 ^a^	2.76 ^a^	4.54 ^a^	6.21	7.49
Terminal boars (TB)	Pn	1.37 ^b,c^	2.58 ^a^	4.44 ^a^	6.17 ^a,b^	7.53 ^a,c^
	LW_SL_	1.38 ^b,d^	2.81 ^b^	4.68 ^b^	6.38 ^b^	7.76 ^b,c^
	D	1.30 ^c^	3.10 ^c^	5.00 ^c^	7.00 ^c^	8.06 ^b^
	D × LW_SL_	1.50 ^a^	2.74 ^b^	4.30 ^a,d^	5.95 ^a^	7.30 ^a^
	LW_SL_ × Pn	1.44 ^a,d^	2.51 ^a^	4.19 ^d^	5.94 ^a^	7.37 ^a^
	D × Pn	1.34 ^b,c^	2.6 ^a^	4.38 ^a^	6.12 ^a^	7.35 ^a^
**Effect of Interaction between the Factors**						
1	Pn	1.30 ^c^	2.57 ^b,c^	4.48 ^c^	6.43 ^b^	7.73 ^b^
	LW_SL_	1.33 ^c^	3.08 ^a^	4.91 ^a,b^	6.49 ^b^	7.81 ^a,b^
	D	1.38 ^b,c^	3.30 ^a^	5.25 ^a^	7.26 ^a^	8.35 ^a^
	D × LW_SL_	1.57 ^a^	2.61 ^b^	3.96 ^d^	5.34 ^d^	6.54 ^e^
	LW_SL_ × Pn	1.57 ^a^	2.47 ^c^	4.08 ^d^	5.85 ^c,d^	7.38 ^c,d^
	D × Pn	1.22 ^d^	2.41 ^c^	4.29 ^c,d^	6.24 ^b,c^	7.54 ^b,c^
2	Pn	1.30 ^c^	2.52 ^b,c^	4.34 ^cd^	6.14 ^b,c^	7.59 ^b,c^
	LW_SL_	1.37 ^c^	2.91 ^a,b^	4.75 ^b^	6.48 ^b^	7.86 ^a,b^
	D	1.29 ^c^	3.01 ^a^	4.92 ^a,b^	7.08 ^a^	8.04 ^a^
	D × LW_SL_	1.51 ^a^	2.91 ^a,b^	4.52 ^b,c^	6.29 ^b,c^	7.69 ^b,c^
	LW_SL_ × Pn	1.36 ^c^	2.77 ^b^	4.33 ^c,d^	5.94 ^c^	7.44 ^c^
	D × Pn	1.53 ^a^	2.61 ^b^	4.46 ^c^	6.19 ^b,c^	7.29 ^cd^
3	Pn	1.26 ^c^	2.64 ^b^	4.47 ^c^	6.27 ^b,c^	7.70 ^b^
	LW_SL_	1.38 ^b,c^	2.56 ^b,c^	4.50 ^b,c^	6.32 ^b,c^	7.79 ^b^
	D	1.22 ^d^	2.86 ^a,b^	4.71 ^b^	6.75 ^b^	7.73 ^b^
	D × LW_SL_	1.44 ^b^	2.64 ^b,c^	4.43 ^c^	6.12 ^c^	7.54 ^b,c^
	LW_SL_ × Pn	1.41 ^b^	2.36 ^c^	4.11 ^d^	5.79 ^cd^	7.12 ^d^
	D × Pn	1.31 ^c^	2.59 ^b,c^	4.34 ^cd^	6.14 ^c^	7.39 ^cd^
4	Pn	1.60 ^a^	2.61 ^b^	4.45 ^c^	5.82 ^cd^	7.10 ^d^
	LW_SL_	1.43 ^b^	2.70 ^b^	4.54 ^bc^	6.22 ^bc^	7.56 ^bc^
	D	1.29 ^c^	3.22 ^a^	5.13 ^ab^	7.08 ^a^	8.13 ^a^
	D × LW_SL_	1.48 ^a,b^	2.81 ^b^	4.43 ^c^	6.06 ^c^	7.41 ^c^
	LW_SL_ × Pn	1.42 ^b^	2.46 ^c^	4.25 ^c,d^	6.17 ^c^	7.54 ^b,c^
	D × Pn	1.29 ^c,d^	2.78 ^b^	4.44 ^c^	5.90 ^c,d^	7.17 ^d^
RMSE		0.28	0.39	0.64	0.96	1.18
*p*-value	P	**	**	*	NS	NS
	TB	**	**	**	**	**
	P × TB	**	**	**	**	**

Pn: Pietrain. LW_SL_: Large White_sireline_. D: Duroc. D × LW_SL_: Duroc × Large White_sireline_. LW_SL_ × Pn: Large White_sireline_ × Pietrain. D × Pn: Duroc × Pietrain. Results of the variance analysis are indicated as significant (* *p* ≤ 0.05, ** *p* ≤ 0.01) or not significant (NS). ^a,b,c,d^: Mean values in the same column marked with a different superscript indicate statistical significance (*p* ≤ 0.05). Mean values with no superscript are not significantly different from any other values. RMSE: root mean square error.

**Table 6:** The effect of terminal boar and parity of sows on average daily gain of piglets.

Factor	Item	Average Daily Gain (g/day)				
		Birth–Day 7	Day 7–14	Day 15–21	Day 22–28	Birth–Weaning
**Effect of Individual Factors**						
Parity (P)	1	194.18 ^a^	251.14 ^a^	253.36 ^a,c^	184.40 ^a^	220.67 ^a^
	2	200.98 ^a^	252.28 ^a^	253.45 ^a^	189.28 ^a^	223.99 ^a^
	3	175.88 ^b^	257.19 ^a^	260.70 ^b,c^	187.91 ^a^	220.18 ^a^
	4	197.41 ^a^	253.77 ^a^	237.93 ^a^	182.72 ^a^	218.02 ^a^
Terminal boars (TB)	Pn	172.10 ^a^	265.11 ^ac^	247.17 ^a^	194.86 ^a^	219.63 ^a,c^
	LW_SL_	204.44 ^b^	266.67 ^ac^	242.47 ^a^	197.26 ^a^	227.68 ^b,c^
	D	245.77 ^c^	272.00 ^c^	285.75 ^b^	151.56 ^c^	238.67 ^b^
	D × LW_SL_	194.34 ^b^	223.19 ^d^	235.64 ^a^	192.32 ^a,b^	211.32 ^a^
	LW_SL_ × Pn	161.79 ^a^	239.84 ^b^	249.34 ^a^	204.39 ^a^	213.82 ^a^
	D × Pn	174.25 ^a^	254.77 ^a,b^	247.79 ^a^	176.08 ^b^	213.17 ^a^
**Effect of Interaction between the Factors**						
1	Pn	170.33 ^b^	272.89 ^a^	279.14 ^a^	185.58 ^a,b^	226.88 ^a,b^
	LW_SL_	242.18 ^a^	262.12 ^a^	225.90 ^b,c^	188.86 ^a,b^	229.68 ^a,b^
	D	274.37 ^a^	279.10 ^a^	286.33 ^a^	155.79 ^b^	248.82 ^a^
	D × LW_SL_	175.20 ^b^	193.49 ^b^	197.06 ^c^	172.28 ^b^	184.39 ^b^
	LW_SL_ × Pn	155.22 ^b,c^	231.19 ^b^	253.09 ^a,b^	217.78 ^a^	214.21 ^b^
	D × Pn	147.80 ^c^	268.05 ^a^	278.65 ^a^	186.12 ^a,b^	220.07 ^a,b^
2	Pn	162.25 ^b,c^	260.51 ^a^	257.06 ^a,b^	206.97 ^a^	221.72 ^ab^
	LW_SL_	217.83 ^a,b^	264.16 ^a^	245.84 ^b^	197.44 ^a^	231.32 ^a^
	D	233.41 ^a,b^	271.94 ^a^	288.00 ^a^	158.96 ^b^	237.99 ^a^
	D × LW_SL_	218.62 ^a,b^	229.50 ^b^	252.03 ^a,b^	202.72 ^a^	225.48 ^a,b^
	LW_SL_ × Pn	198.09 ^b^	222.88 ^b^	230.08 ^b,c^	213.94 ^a^	216.25 ^a,b^
	D × Pn	175.67 ^b^	264.70 ^a^	247.67 ^b^	157.15 ^b^	211.18 ^b^
3	Pn	181.23 ^b^	263.73 ^a^	257.74 ^a,b^	203.84 ^a^	225.72 ^a,b^
	LW_SL_	169.10 ^b,c^	276.95 ^a^	258.84 ^a,b^	210.89 ^a^	228.94 ^a,b^
	D	212.06 ^ab^	264.86 ^a^	290.18 ^a^	145.41 ^b^	226.72 ^a,b^
	D × LW_SL_	179.43 ^b^	238.05 ^a,b^	260.46 ^a,b^	202.72 ^a^	220.05 ^a,b^
	LW_SL_ × Pn	139.98 ^c^	249.61 ^a,b^	240.34 ^b^	190.15 ^a,b^	204.91 ^b^
	D × Pn	173.50 ^b^	249.94 ^a,b^	256.63 ^a,b^	179.41 ^a,b^	214.74 ^b^
4	Pn	174.59 ^b^	261.31 ^a^	194.73 ^c^	183.05 ^a,b^	204.22 ^b^
	LW_SL_	188.65 ^b^	263.44 ^a^	239.29 ^b^	191.84 ^a^	220.78 ^a,b^
	D	263.22 ^a^	272.09 ^a^	278.49 ^a^	151.09 ^b^	241.14 ^a^
	D × LW_SL_	204.12 ^b^	231.71 ^b^	232.99 ^b^	193.06 ^a^	215.35 ^b^
	LW_SL_ × Pn	153.86 ^c^	255.69 ^a,b^	273.84 ^a^	195.65 ^a^	219.90 ^a,b^
	D × Pn	200.02 ^b^	236.38 ^a,b^	208.20 ^b,c^	181.62 ^a,b^	206.70 ^b^
RMSE		55.26	59.71	78.79	66.88	42.27
*p*-value	P	**	NS	**	NS	NS
	TB	**	**	**	**	**
	P × TB	**	**	**	**	**

Pn: Pietrain. LW_SL_: Large White_sireline_. D: Duroc. D × LW_SL_: Duroc × Large White_sireline_. LW_SL_ × Pn: Large White_sireline_ × Pietrain. D × Pn: Duroc × Pietrain. Results of the variance analysis are indicated as significant (** *p* ≤ 0.01) or not significant (NS). ^a,b,c,d^: Mean values in the same column marked with a different superscript indicate statistical significance (*p* ≤ 0.05). Mean values with no superscript are not significantly different from any other values. RMSE: root mean square error.

## Data Availability

The data presented in this study are available on request from the corresponding author. The data are not publicly available to preserve privacy of the data.
